# Supplementary material for: Potentiating hypoxic microenvironment for antibiotic activation by photodynamic therapy to combat bacterial biofilm infections
Source: Nat Commun. 2022 Jul 5;13:3875. doi: 10.1038/s41467-022-31479-x (PMC9256606; doi:10.1038/s41467-022-31479-x)
Supplement: Supplementary file 1 — Supplementary Information [file 41467_2022_31479_MOESM1_ESM.pdf]

Supplementary Information

Potentiating hypoxic microenvironment for antibiotic activation by photodynamic therapy to combat bacterial biofilm infections

Weijun Xiu[1], Ling Wan[1], Kaili Yang[1], Xiao Li[1], Lihui Yuwen[1,*], Heng Dong[2], Yongbin Mou[2], Dongliang Yang[3], and Lianhui Wang[1,*]

[1]State Key Laboratory for Organic Electronics and Information Displays & Jiangsu Key Laboratory for Biosensors, Institute of Advanced Materials (IAM), Jiangsu National Synergetic Innovation Centre for Advanced Materials (SICAM), Nanjing University of Posts and Telecommunications, Nanjing 210023, China

[2]Nanjing Stomatological Hospital, School of Medicine, Nanjing University, Nanjing, 210008, China

[3]School of Physical and Mathematical Sciences & Institute of Advanced Materials (IAM), Nanjing Tech University, Nanjing 211800, China

[*]Correspondence and requests for materials should be addressed to L. Y. (email: iamlhyuwen@njupt.edu.cn) and L. W. (email: iamlhwang@njupt.edu.cn).

Supplementary Methods

***In vitro* antibacterial activity of MNZ under different oxygen levels.** Methicillin-resistant *Staphylococcus aureus* (MRSA, ATCC43300) were grown in chemically defined medium under shaking at 220 rpm and 37°C over 12 h and further diluted to 10^7 CFU/mL with chemically defined medium[1]. The MRSA suspension was mixed with different concentrations of MNZ and incubated under different oxygen levels. The antibacterial efficiency of MNZ was evaluated by using plate counting method.

Penetration of MNZ in MRSA biofilms. Firstly, the MRSA biofilm was growth in Transwell inserts for 48 h. Then, PBS dispersions of HCM NPs (Ce6: 50 μg/mL, MNZ: 25 μg/mL) were transferred into the inserts and incubated with MRSA biofilms for different times, and irradiated with 635 nm laser (20 mW/cm^2, 30 min) for PDT group. The amount of MNZ penetrated into receiver plate was calculated by using UV-vis absorption spectroscopy (UV-3600, Shimadzu, Japan).

Quantitative real-time PCR. The RNA was extracted from MRSA under different experimental conditions by using bacteria total RNA isolation kit (Sangon Biotech, China). The complementary DNA (cDNA) was further synthesized by using a reverse transcription kit (Takara) as per the instructions. The expression levels of genes encoding fibronectin binding protein B (*fnbB*), elastin binding protein (*ebps*), and intercellular adhesion biofilm required genes type C and D (*icaC and icaD*) were detected by using the synergy brands (SYBR) Green Select Master Mix (Bio-Rad) in triplicate on an AbiPrism 7900 HT cycler (Applied Biosystems), with 16sRNA as a normalized gene. The fold change of the target mRNA relative to the controls was determined using the $2^{-\Delta\Delta CT}$ method. All primers used in qPCR were designed according to previous report and were listed in Supplementary Table 1[2].

Wound blotting for MRSA biofilm detection. The nitrocellulose membrane was pressed to the infected tissues of mice for 10 seconds and left for natural drying. After stained with ruthenium red (5 mg/mL) for 1 min, the membrane was washed three times by soaking in a mixed solution (containing 40% methanol 10% acetic acid) for 30 minutes to decrease the amount of nonspecifically bound staining solution[3]. The stained membrane was then photographed.

Supplementary Figures

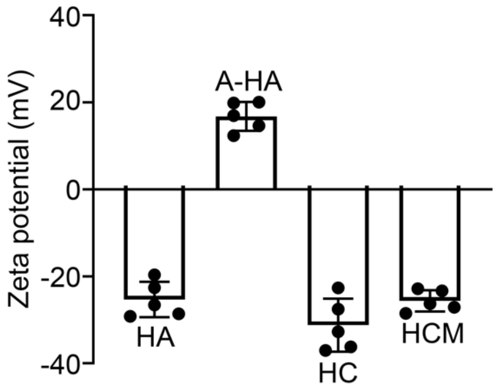

Supplementary Fig. 1 Zeta potential of HA, A-HA, HC NPs, and HCM NPs (n = 5 independent samples; mean ± SD). Source data are provided as a Source Data file.

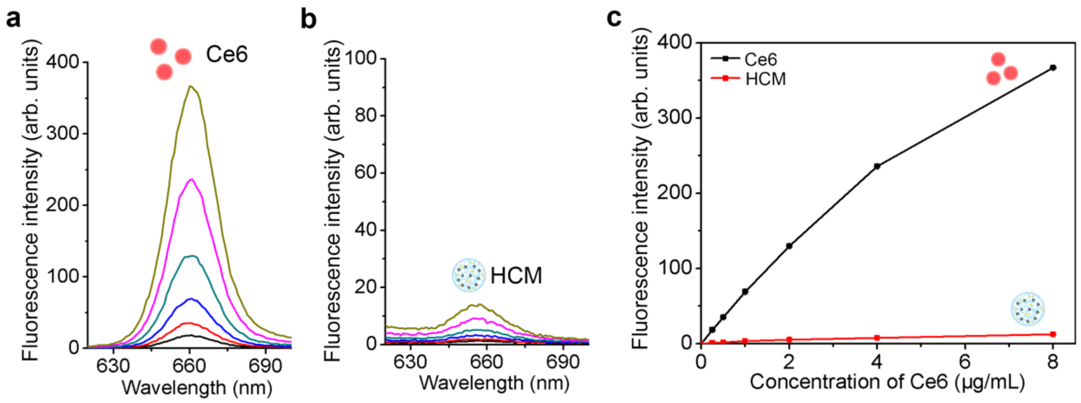

Supplementary Fig. 2 Aggregation induced fluorescence quenching of Ce6 in HCM NPs. **a,b,** Fluorescence intensity of Ce6 (**a**) and HCM NPs (**b**) at different concentrations (from bottom to top, Ce6: 0.2, 0.5, 1, 2, 4, 8 μg/mL). **c,** Fluorescence intensity of Ce6 and HCM NPs dispersions at different concentrations. Source data are provided as a Source Data file.

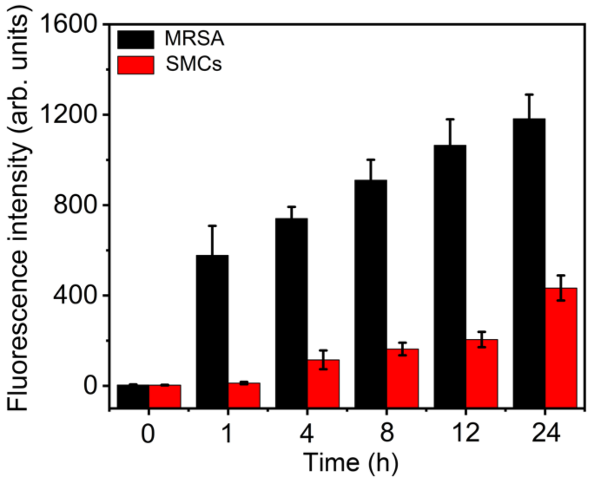

Supplementary Fig. 3 Fluorescence intensity of HCM NPs solutions (Ce6: 20 μg/mL) after incubated with MRSA biofilms and mice smooth muscle cells (SMCs) for different times (n = 20 biologically independent samples; mean ± SD). Source data are provided as a Source Data file.

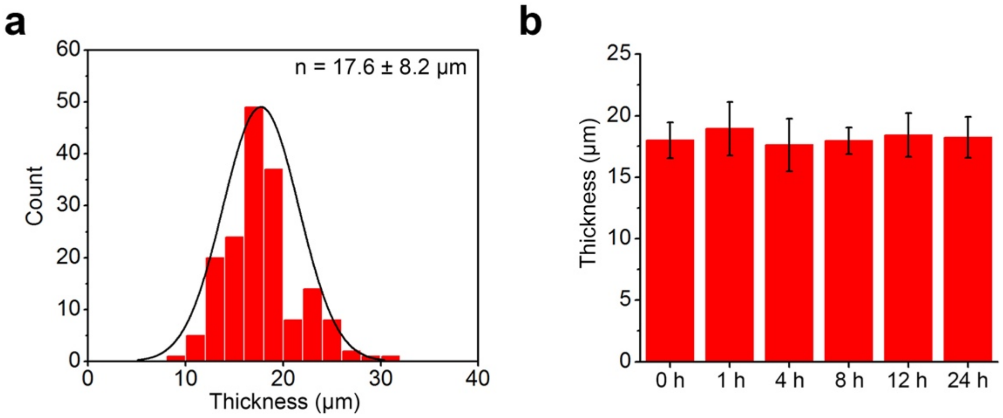

Supplementary Fig. 4 Thickness of MRSA biofilms. **a,** Thickness of MRSA biofilms without any treatment (n=200). **b,** Thickness of MRSA biofilms after incubation with HCM NPs for different times (n = 20 biologically independent samples; mean ± SD). Source data are provided as a Source Data file.

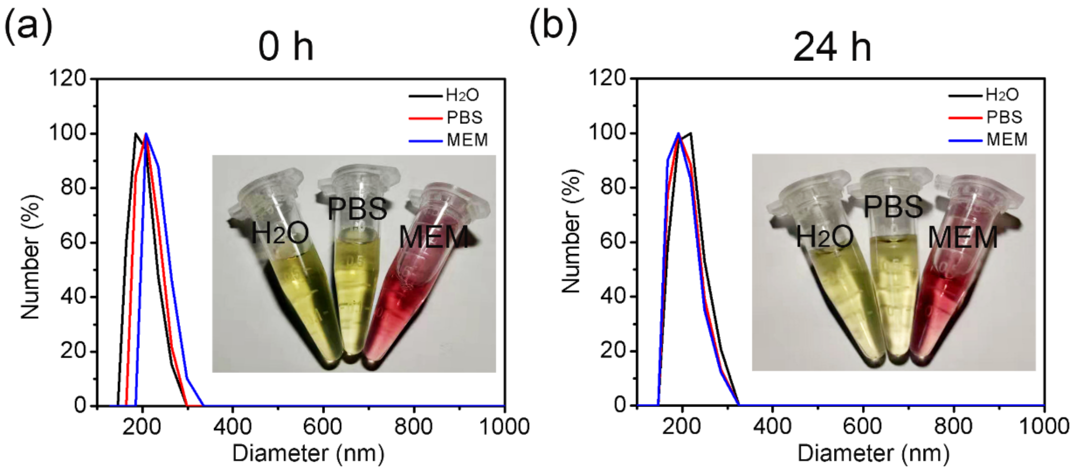

Supplementary Fig. 5 Colloidal stability of HCM NPs. **a,b,** Hydrodynamic sizes and photographs (inset) of HCM NPs (Ce6: 20 μg/mL; MNZ: 10 μg/mL) dispersed in different mediums (H_2O, PBS, and minimum Eagle's medium (MEM)) for 0 h (**a**) and 24 h (**b**). Source data are provided as a Source Data file.

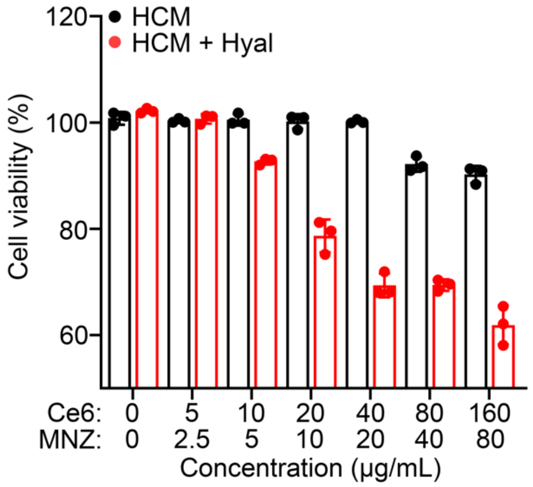

Supplementary Fig. 6 Viability of human normal liver (L-O2) cells incubated with HCM NPs or HCM NPs + Hyal (100 unit/mL) for 24 h (n = 3 biologically independent samples; mean ± SD). Source data are provided as a Source Data file.

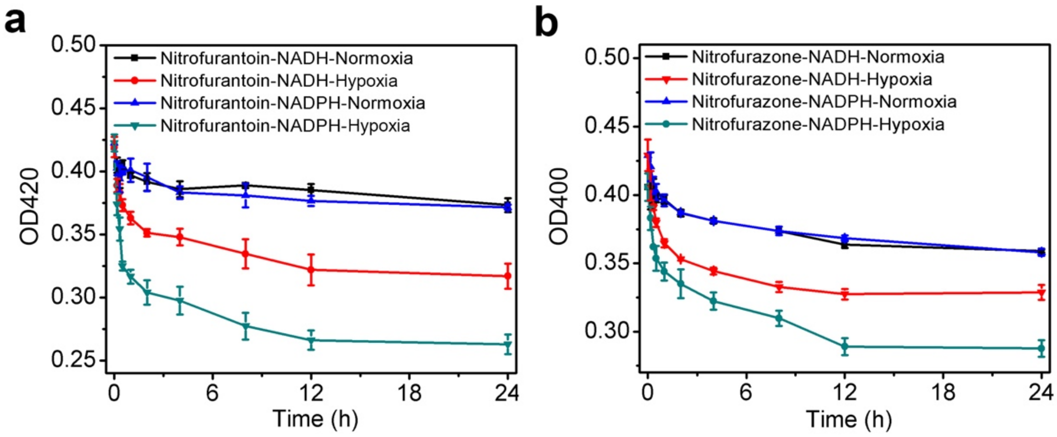

Supplementary Fig. 7 Characterization of nitroreductase activity from MRSA under different conditions. **a,b,** Absorbance of nitrofurantoin (**a**) and nitrofurazone (**b**) at 420 nm (OD420) and 400 nm (OD400), respectively, after incubated with cell extracts of MRSA biofilms in normoxic condition and hypoxic condition (5% O_2, 95% CO_2) for different times (n = 3 biologically independent samples; mean ± SD). Source data are provided as a Source Data file.

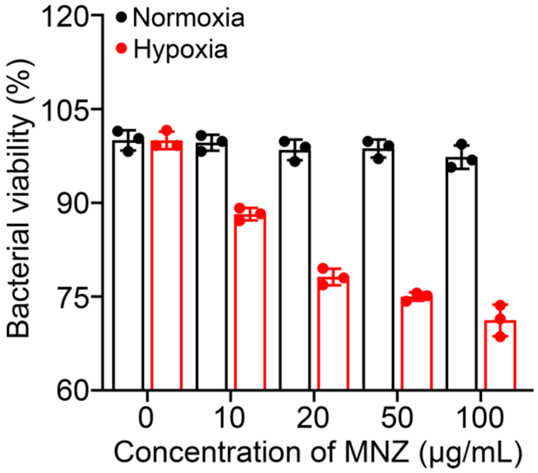

Supplementary Fig. 8 Bacterial viability of MRSA after incubated with MNZ for 12 h under normoxic condition and hypoxic (5% O_2, 95% CO_2) condition (n = 3 biologically independent samples; mean ± SD). Source data are provided as a Source Data file.

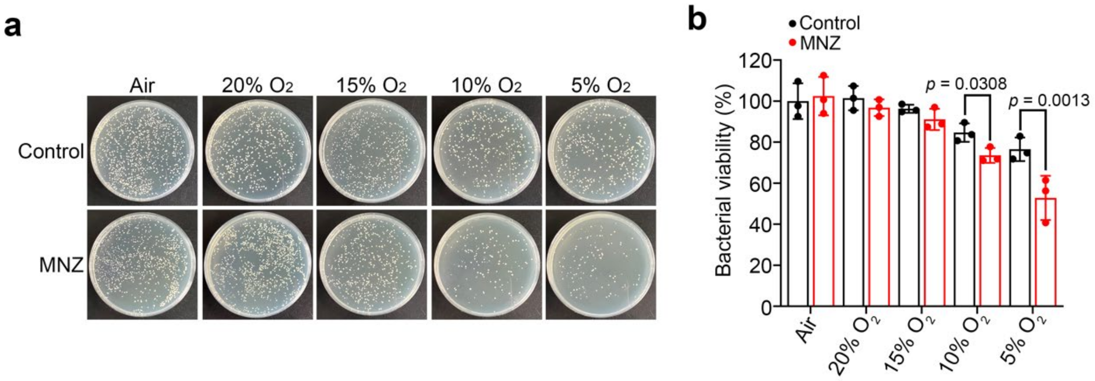

Supplementary Fig. 9 Bacterial inactivation efficiency of MNZ under different O_2 levels. **a,** Photographs of the MRSA colony grown on LB plates after incubated without or with MNZ (50 μg/mL) for 24 h under different O_2 levels. **b,** Bacterial viability of MRSA after incubated without or with MNZ (50 μg/mL) under different O_2 levels (n = 3 biologically independent samples; mean ± SD). Statistical significance was analyzed via two-way ANOVA with a Tukey post-hoc test. Source data are provided as a Source Data file.

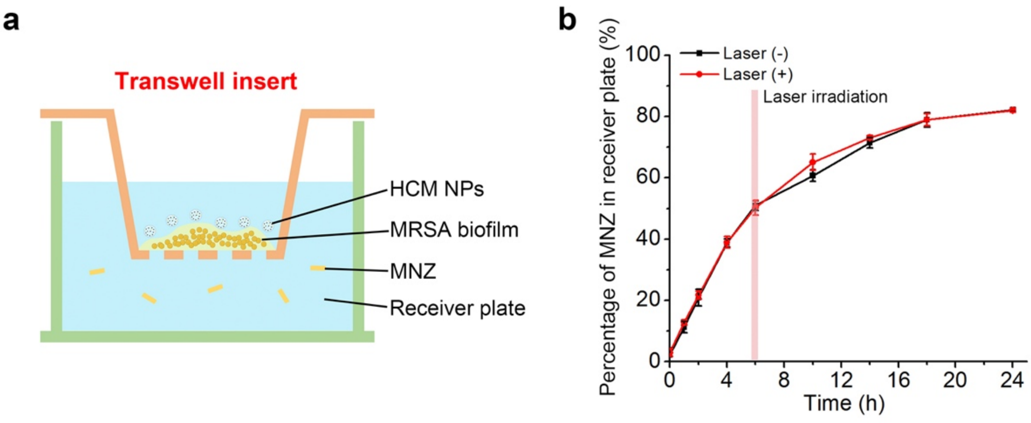

Supplementary Fig. 10 Penetration of MNZ within MRSA biofilms. **a,** Schematic illustration for the penetration of MNZ in MRSA biofilms grown on Transwell inserts after incubation with HCM NPs. **b,** Percentage of MNZ from HCM NPs penetrated within MRSA biofilms with or without laser irradiation (635 nm, 20 mW/cm^2, 30 min) for different times (n = 3 biologically independent samples; mean ± SD). Source data are provided as a Source Data file.

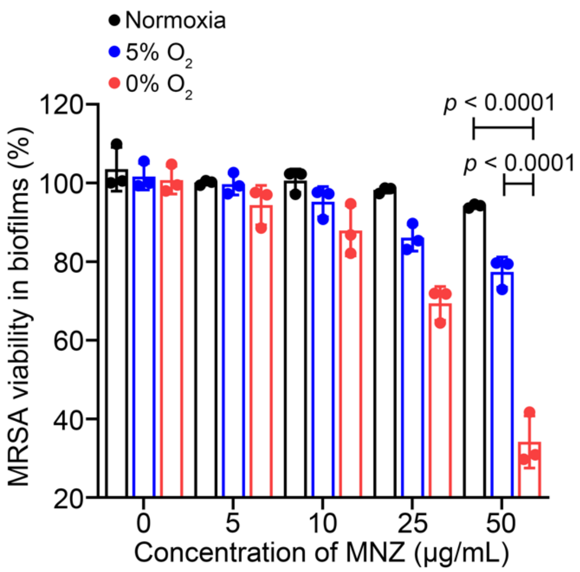

Supplementary Fig. 11 Viability of MRSA in biofilms after incubated with different concentrations of MNZ under different O_2 levels (n = 3 biologically independent samples; mean ± SD). Statistical significance was analyzed via two-way ANOVA with a Tukey post-hoc test. Source data are provided as a Source Data file.

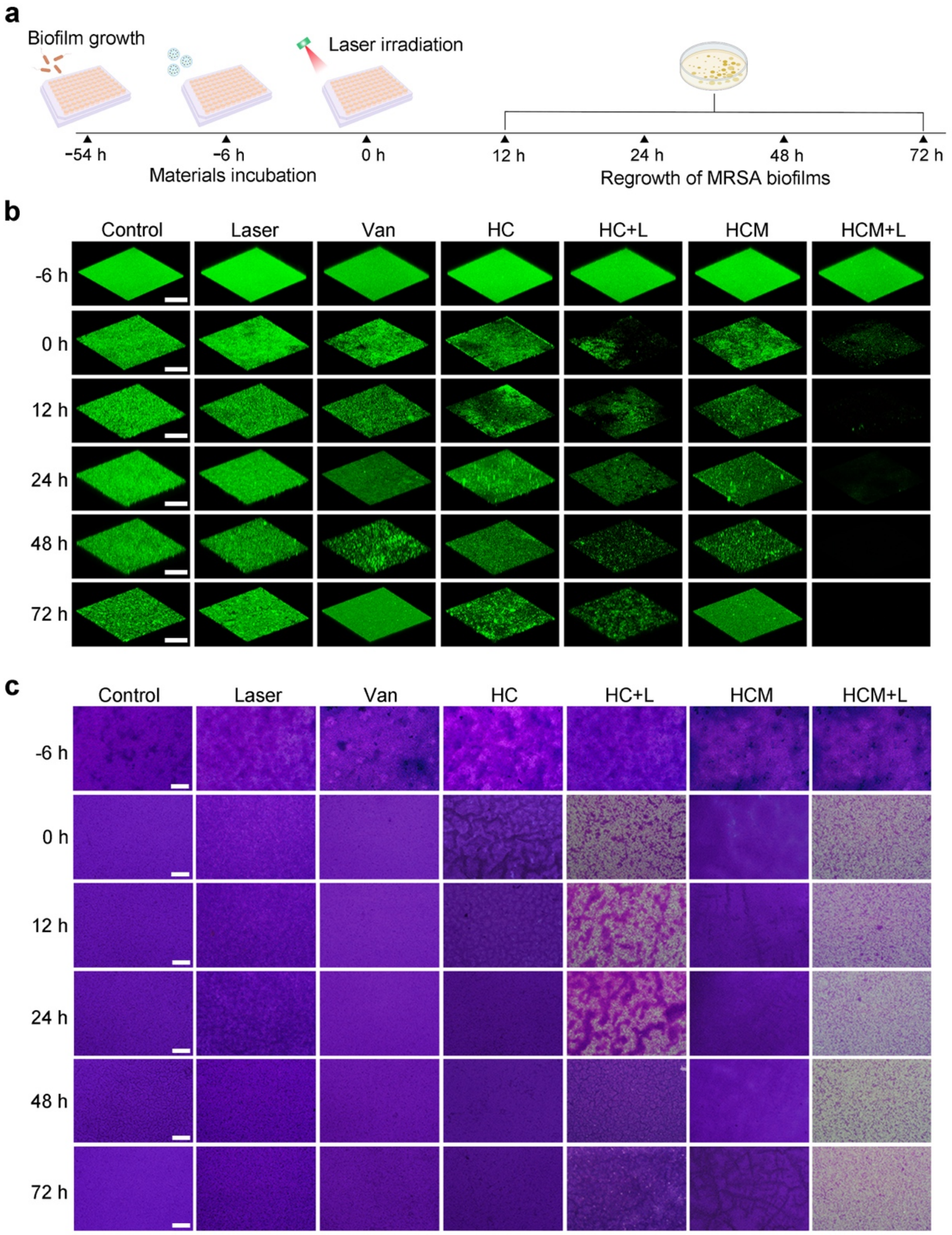

Supplementary Fig. 12 Long-term inhibition of MRSA biofilms treated by HCM NPs *in vitro*. **a,** Schematic illustration of the experimental procedure. **b,** 3D CLSM images of MRSA biofilms stained by Calcein-AM (green) after various treatments. Scale bar is 200 μm. The size of CLSM images is 630 μm × 630 μm. **c,** Micrographs of MRSA biofilms stained by crystal violet after various treatments. Scale bar is 200 μm. Three independent experiments were performed and representative results are shown in **b** and **c**.

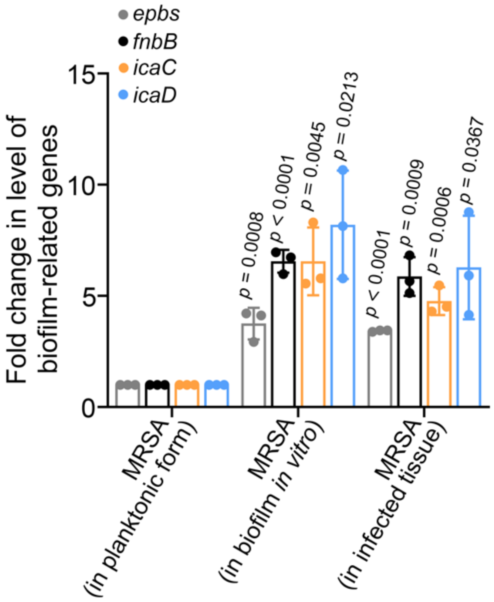

Supplementary Fig. 13 Expression levels of *ebps*, *fnbB*, *icaC*, and *icaD* transcripts from MRSA in biofilm *in vitro* and infected tissues versus planktonic MRSA (n = 3 biologically independent samples; mean ± SD). Statistical significance was analyzed via two-way ANOVA with a Tukey post-hoc test. Source data are provided as a Source Data file.

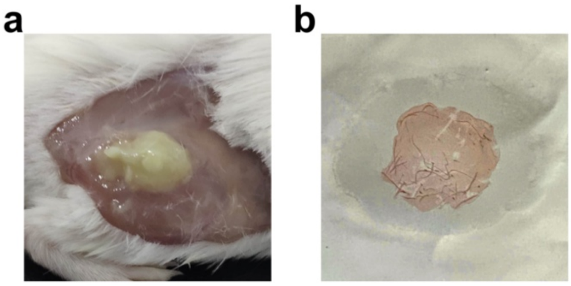

Supplementary Fig. 14 Biofilm detection by using wound blotting method. **a,b,** Photographs of MRSA biofilm infected tissue (**a**) and ruthenium red stained nitrocellulose membrane (**b**) applied with infected tissue.

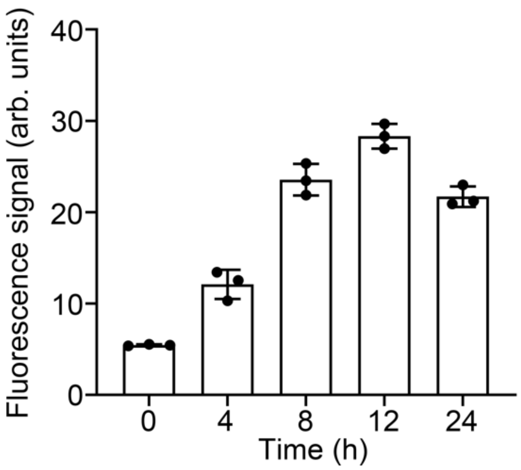

Supplementary Fig. 15 The average fluorescence signal of MRSA biofilm infected sites after mice *i.v.* injected with HCM NPs for different times (n = 3 biologically independent samples; mean ± SD). Source data are provided as a Source Data file.

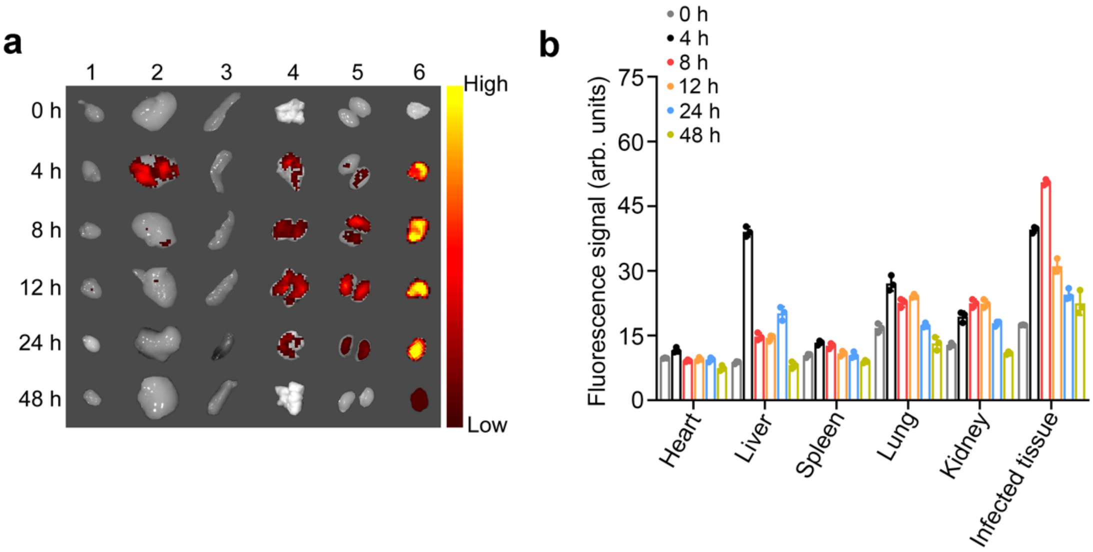

Supplementary Fig. 16 Retention and distribution of HCM NPs in MRSA biofilm infected mice after *i.v.* injection. **a,b,** Fluorescence images (**a**) and average fluorescence intensity (**b**) of mice major organs (heart (1), liver (2), spleen (3), lung (4), kidney (5)) and biofilm infected tissues (6) after *i.v.* injection of HCM NPs (Ce6 = 4 mg/kg; MNZ = 2 mg/kg) at different times (n = 3 biologically independent samples; mean ± SD). Source data are provided as a Source Data file.

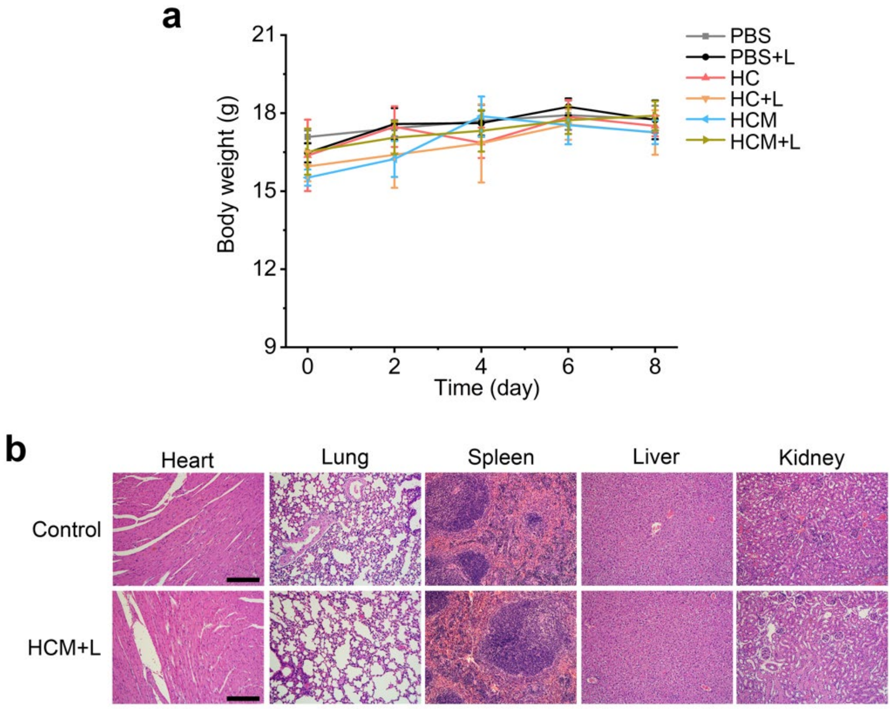

Supplementary Fig. 17 Toxicity evaluation of HCM NPs to MRSA biofilm infected mice with laser irradiation. **a,** Body weights of the mice after various treatments (n = 5 biologically independent animals; mean ± SD). **b,** Micrographs of H&E stained slices of major organs (heart, liver, spleen, lung, and kidney) of mice after treatments with PBS (Control) and HCM NPs with laser irradiation (HCM+L) at 8 d post-treatment. Scale bar is 200 μm. Source data are provided as a Source Data file.

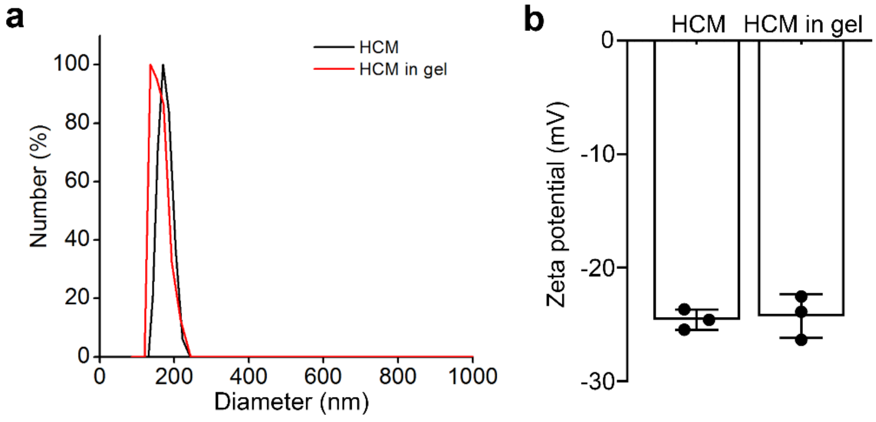

Supplementary Fig. 18 Hydrodynamic sizes and zeta potential of HCM NPs at different conditions. **a,b,** Hydrodynamic sizes (**a**) and zeta potential (**b**) of free HCM NPs and HCM NPs in sprayed gel (n = 3 independent samples; mean ± SD). Source data are provided as a Source Data file.

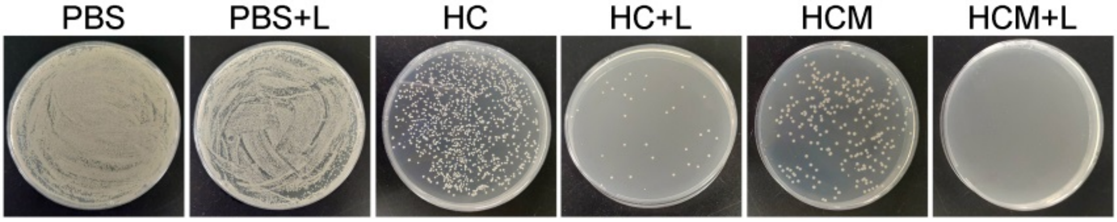

Supplementary Fig. 19 Photographs of the MRSA colony grown on LB plates from PBS dispersions of biofilm infected tissues at 12 d post-treatment.

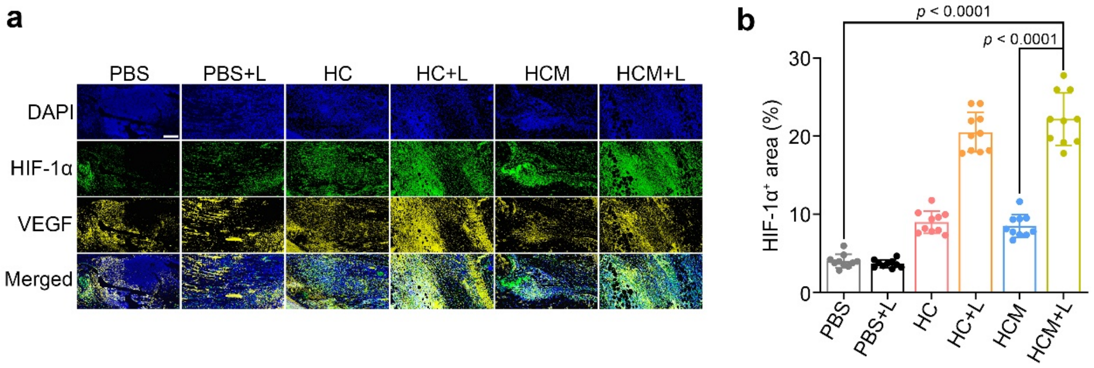

Supplementary Fig. 20 Hypoxic condition of MRSA biofilm infected wounds in diabetic mice. **a,** Immunofluorescence images of HIF-1α (green) and VEGF (yellow) in infected wounds at 4 d post-treatment. **b,** Percentage of HIF-1α$^+$ area in immunofluorescence images (n = 10 biologically independent samples; mean ± SD). Scale bar is 200 μm. Statistical significance was analyzed via one-way ANOVA with a Tukey post-hoc test. Source data are provided as a Source Data file.

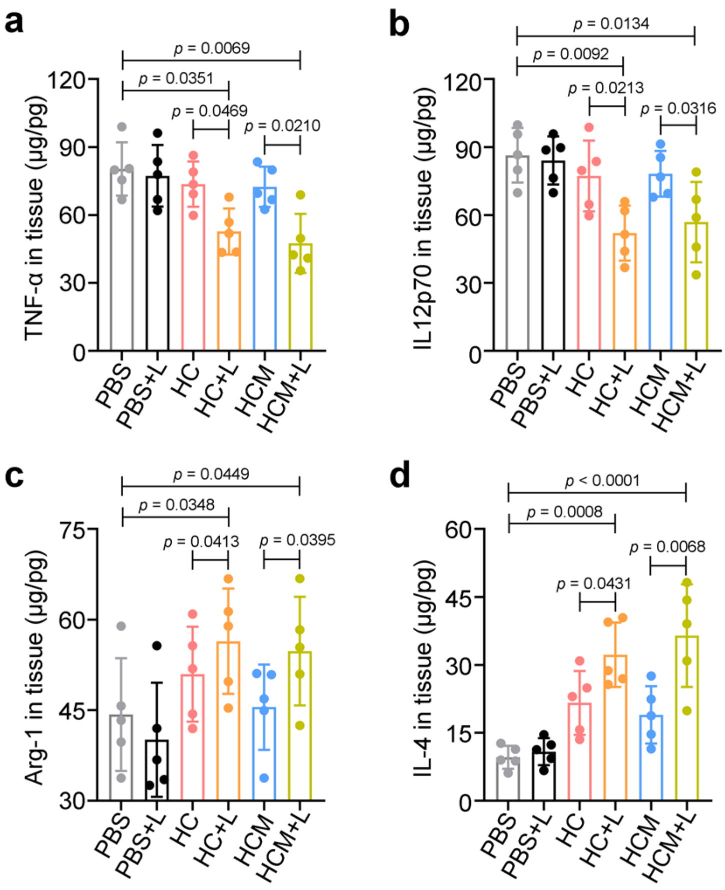

Supplementary Fig. 21 Secretion levels of TNF-α (**a**), IL-12p70 (**b**), Arg-1 (**c**), and IL-4 (**d**) in infected tissues at 4 d post-treatment (n = 5 biologically independent samples; mean ± SD). Statistical significance was analyzed via one-way ANOVA with a Tukey post-hoc test. Source data are provided as a Source Data file.

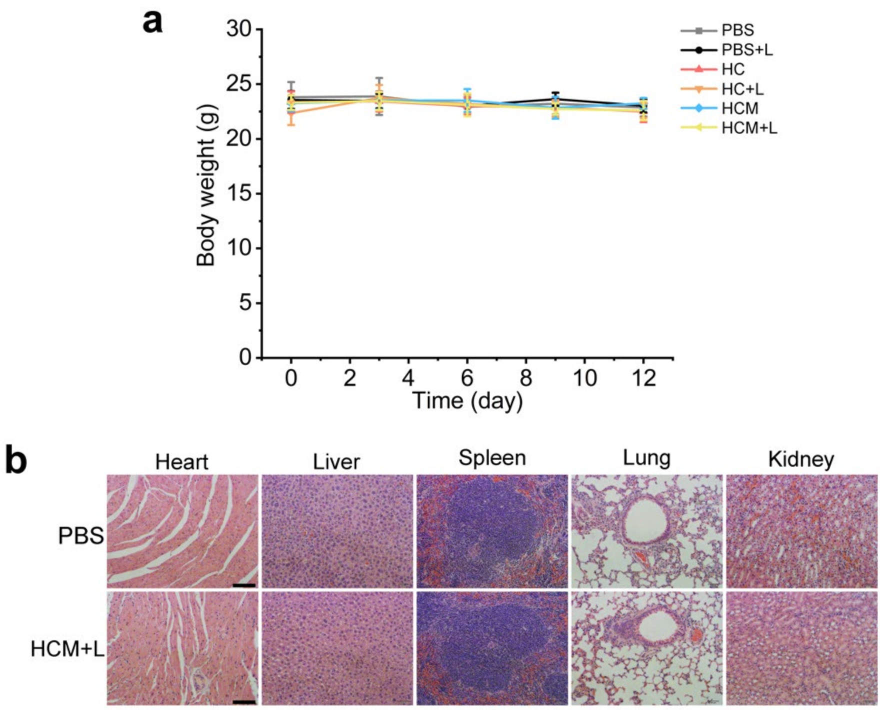

Supplementary Fig. 22 Toxicity evaluation of the HCM gel to biofilm infected diabetic mice. **a,** Body weights of the mice at 12 d post-treatment (n = 5 biologically independent animals; mean ± SD). **b,** Micrographs of H&E stained slices of major organs (heart, liver, spleen, lung, and kidney) after treatments with PBS (Control) and HCM NPs with laser irradiation (HCM+L) at 12 d post-treatment. Scale bar is 100 μm. Source data are provided as a Source Data file.

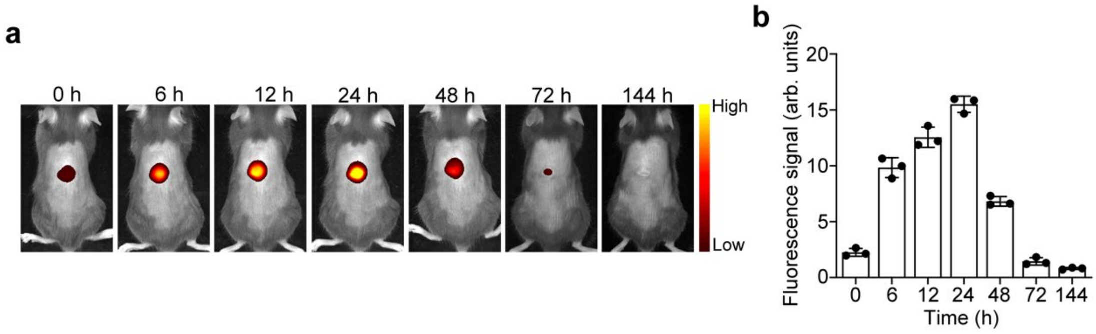

Supplementary Fig. 23 Fluorescence imaging of biofilm infected wound in mice after treated with HCM gel. **a,b,** Fluorescence images (**a**) and fluorescence intensity (**b**) of biofilm infected wound in mice after treated with HCM gel for different times (n = 3 biologically independent animals; mean ± SD). Source data are provided as a Source Data file.

Supplementary Table 1 Sequences of oligonucleotide primers used for qPCR[2].

Genes	Nucleotide sequence of primers (5'-3')	Accession numbers	Annealing temperature	Amplicon size (bp)
epbs	5-GGTGCAGCTGGTGCAATGGGTGT-3<br>5-GCTGCGCCTCCAGCCAAACCT-3	U48826.2	60°C	191
fnbB	5-ACGCTCAAGGCGACGGCAAAG-3<br>5-ACCTTCTGCATGACCTTCTGCACCT-3	X62992.1	60°C	197
icaC	5-CTTGGGTATTTGCACGCATT-3<br>5-GCAATATCATGCCGACACCT-3	AF086783	60°C	209
icaD	5-ACCCAACGCTAAAATCATCG-3<br>5-GCGAAAATGCCCATAGTTTC-3	AF086783	60°C	211
**16sRNA**	5-GGGACCCGCACAAGCGGTGG-3<br>5-GGGTTGCGCTCGTTGCGGGA-3	L37597.1	60°C	191

Supplementary References

1. Hussain, M., Hastings, J. & White, P. A chemically defined medium for slime production by coagulase-negative staphylococci. *J. Med. Microbiol.* **34**, 143 (1991).

2. Atshan, SS. et al. Quantitative PCR analysis of genes expressed during biofilm development of methicillin resistant *Staphylococcus aureus* (MRSA). *Infect., Genet. Evol.* **18**, 106-112 (2013).

3. Nakagami, G. et al. Biofilm detection by wound blotting can predict slough development in pressure ulcers: A prospective observational study. *Wound Repair Regen.* **25**, 131-138 (2017).
