# Supplementary material for: Potentiating hypoxic microenvironment for antibiotic activation by photodynamic therapy to combat bacterial biofilm infections
Source: Nat Commun. 2022 Jul 5;13:3875. doi: 10.1038/s41467-022-31479-x (PMC9256606; doi:10.1038/s41467-022-31479-x)
Supplement: Supplementary file 3 — Reporting Summary [file 41467_2022_31479_MOESM3_ESM.pdf]

nature portfolio

Corresponding author(s): Lianhui Wang and Lihui Yuwen

Last updated by author(s): May 30, 2022

Reporting Summary

Nature Portfolio wishes to improve the reproducibility of the work that we publish. This form provides structure for consistency and transparency in reporting. For further information on Nature Portfolio policies, see our Editorial Policies and the Editorial Policy Checklist.

Statistics

For all statistical analyses, confirm that the following items are present in the figure legend, table legend, main text, or Methods section.

n/a	Confirmed	
☐	☒	The exact sample size (n) for each experimental group/condition, given as a discrete number and unit of measurement
☐	☒	A statement on whether measurements were taken from distinct samples or whether the same sample was measured repeatedly
☐	☒	The statistical test(s) used AND whether they are one- or two-sided<br>*Only common tests should be described solely by name; describe more complex techniques in the Methods section.*
☐	☒	A description of all covariates tested
☒	☐	A description of any assumptions or corrections, such as tests of normality and adjustment for multiple comparisons
☐	☒	A full description of the statistical parameters including central tendency (e.g. means) or other basic estimates (e.g. regression coefficient) AND variation (e.g. standard deviation) or associated estimates of uncertainty (e.g. confidence intervals)
☐	☒	For null hypothesis testing, the test statistic (e.g. F, t, r) with confidence intervals, effect sizes, degrees of freedom and P value noted<br>*Give P values as exact values whenever suitable.*
☒	☐	For Bayesian analysis, information on the choice of priors and Markov chain Monte Carlo settings
☒	☐	For hierarchical and complex designs, identification of the appropriate level for tests and full reporting of outcomes
☒	☐	Estimates of effect sizes (e.g. Cohen's d, Pearson's r), indicating how they were calculated

Our web collection on statistics for biologists contains articles on many of the points above.

Software and code

Policy information about availability of computer code

Data collection	FV10-ASW software (version 3.0) for Confocal images, Gel-Pro Analyzer (version 6.0) software for Western blot, IVIS Lumina software for fluorescence images.
Data analysis	Data mean ± standard deviation (SD) were calculated by Excel, Data Statistical analyses were performed by Graphpad Prism (version 8.4.0).

For manuscripts utilizing custom algorithms or software that are central to the research but not yet described in published literature, software must be made available to editors and reviewers. We strongly encourage code deposition in a community repository (e.g. GitHub). See the Nature Portfolio guidelines for submitting code & software for further information.

Data

Policy information about availability of data

All manuscripts must include a data availability statement. This statement should provide the following information, where applicable:

- Accession codes, unique identifiers, or web links for publicly available datasets
- A description of any restrictions on data availability
- For clinical datasets or third party data, please ensure that the statement adheres to our policy

All the data supporting the findings of this study are available within the article and its Supplementary information files and from the corresponding author upon request.

Field-specific reporting

Please select the one below that is the best fit for your research. If you are not sure, read the appropriate sections before making your selection.

☒ Life sciences ☐ Behavioural & social sciences ☐ Ecological, evolutionary & environmental sciences

For a reference copy of the document with all sections, see nature.com/documents/nr-reporting-summary-flat.pdf

Life sciences study design

All studies must disclose on these points even when the disclosure is negative.

Sample size	Sample size were determined according to the pilot and on the basis of the previous experimental experiences (Research, 2020, 2020: 9426453; Science Advances, 2020, 6: eabb0025). Generally three independent replicates were done for in vitro experiments, and five independent biological replicates for in vivo experiments.
Data exclusions	No data were excluded.
Replication	The experience were repeated at least three times, and experimental findings were reproducible. The experiment performed with independent replicates was described in the corresponding figure legends.
Randomization	All samples were randomly allocated into experimental groups.
Blinding	No formal blinding was used for the animal studies. Because blinding has no effect on the experiment results. Unbiased experimental procedure and data analysis were carried out as far as possible.

Reporting for specific materials, systems and methods

We require information from authors about some types of materials, experimental systems and methods used in many studies. Here, indicate whether each material, system or method listed is relevant to your study. If you are not sure if a list item applies to your research, read the appropriate section before selecting a response.

Materials & experimental systems

n/a	Involved in the study	
☐	☒	Antibodies
☐	☒	Eukaryotic cell lines
☒	☐	Palaeontology and archaeology
☐	☒	Animals and other organisms
☒	☐	Human research participants
☒	☐	Clinical data
☒	☐	Dual use research of concern

Methods

n/a	Involved in the study	
☒	☐	ChIP-seq
☒	☐	Flow cytometry
☒	☐	MRI-based neuroimaging

Antibodies

Antibodies used

The following antibodies were used for immunofluorescence imaging:
1) Anti-HIF-1α (Abcam, Cat no: ab16066, Mouse monoclonal [mgc3])
2) Anti-VEGF (Abcam, Cat no: ab52917, Rabbit monoclonal [EP1176Y])
3) Anti-F4/80 (Abcam, Cat no: ab6640, Rat monoclonal [CI:A3-1])
4) Anti-CD80 (Abcam, Cat no: ab254579, Rabbit polyclonal)
5) Anti-CD206 (Abcam, Cat no: ab64693, Rabbit polyclonal)
6) Alexa Fluor594-Goat Anti-Rat IgG (Abcam, ab150160, Goat Polyclonal)
7) FITC-Goat Anti-Rabbit IgG (Jackson ImmunoResearch, 111-095-003, Goat Polyclonal)
8) Tetramethylrhodamine (TRITC)-Goat Anti-Mouse IgG (Jackson ImmunoResearch, 115-025-062, Goat Polyclonal)

The following kit was used for ELISA:
1) Mouse IL-6 ELISA Kit (Abcam, Cat no: ab222503)
2) Mouse IL-4 ELISA Kit (Abcam, Cat no: ab100710)
3) Mouse Arg-1 ELISA Kit (Abcam, Cat no: ab269541)
4) Mouse TGF-β ELISA Kit (Abcam, Cat no: ab119557)
5) Mouse TNF-α ELISA Kit (Abcam, Cat no: ab208348)
6) Mouse IL-12p70 ELISA Kit (Abcam, Cat no: ab119531)

The following primary antibodies were used for Western blotting:

	1) Anti-CD80 (Abcam, Cat no: ab254579, Rabbit polyclonal)<br>2) Anti-CD206 (Abcam, Cat no: ab64693, Rabbit polyclonal)<br>3) Anti-GAPDH (Abcam, Cat no: ab8245, Mouse monoclonal [6C5])
Validation	Anti-HIF-1α antibody (validated by Abcam, ab16066)<br>Anti-VEGF antibody (validated by Abcam, ab52917)<br>Anti-F4/80 antibody (validated by Abcam, ab6640)<br>Anti-CD80 antibody (validated by Abcam, ab254579)<br>Anti-CD206 antibody (validated by Abcam, ab64693)<br>Anti-GAPDH antibody (validated by Abcam, ab8245)<br>Alexa Fluor594-Goat Anti-Rat IgG (validated by Abcam, ab150160)<br>FITC-Goat Anti-Rabbit IgG (validated by Jackson ImmunoResearch, 115-025-062)<br>Tetramethylrhodamine (TRITC)-Goat Anti-Mouse IgG (validated by Jackson ImmunoResearch, 115-025-062)

Eukaryotic cell lines

Policy information about cell lines

Cell line source(s)	Human normal liver cells (L-O2) and mice smooth muscle cells (SMCs) were purchased from KeyGen BioTech.
Authentication	Human normal liver cells (L-O2) and mice smooth muscle cells (SMCs) were authenticated by STR profiling.
Mycoplasma contamination	No mycoplasma contamination was found.
Commonly misidentified lines<br>(See ICLAC register)	No commonly misidentified cell lines were used.

Animals and other organisms

Policy information about studies involving animals; ARRIVE guidelines recommended for reporting animal research

Laboratory animals	Female Balb/c mice (6-8 weeks old) and male C57BL/6 mice (6-8 weeks old) were purchased from Nanjing Junke Biological Engineering Co., Ltd.
Wild animals	The study did not involve wild animals.
Field-collected samples	The study did not involve samples collected from the field.
Ethics oversight	All animal procedures were performed in accordance with the Guidelines for the Care and Use of Laboratory Animals of Nanjing Tech University and approved by the Animal Ethics Committee of Nanjing Tech University (LL-2020-0305-05).

Note that full information on the approval of the study protocol must also be provided in the manuscript.
