# Supplementary figures and images for: Potentiating hypoxic microenvironment for antibiotic activation by photodynamic therapy to combat bacterial biofilm infections
Source: Nat Commun. 2022 Jul 5;13:3875. doi: 10.1038/s41467-022-31479-x (PMC9256606; doi:10.1038/s41467-022-31479-x)

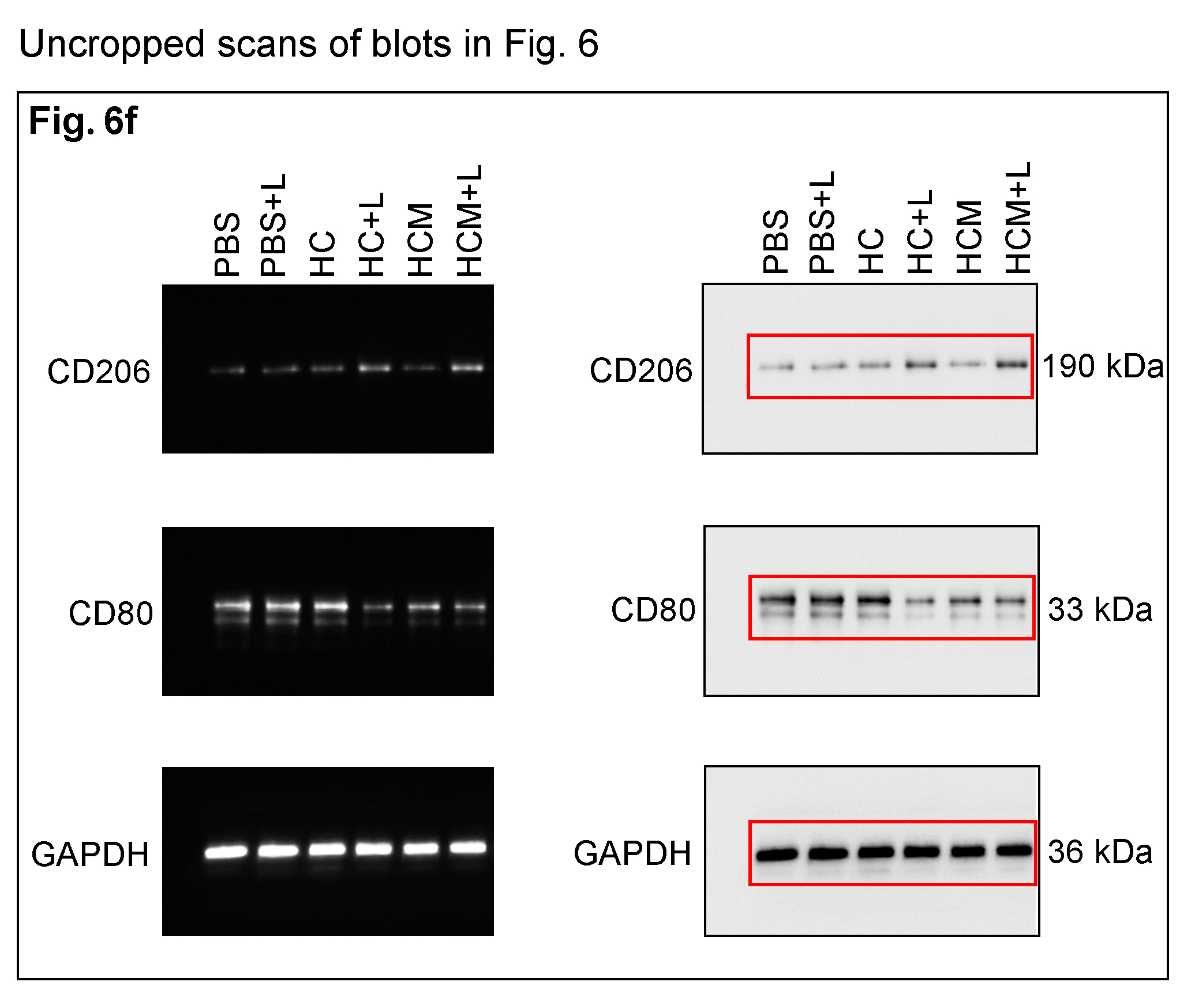

Supplement: Supplementary file 4 — Source Data [file 41467_2022_31479_MOESM4_ESM.zip › Source file/Figure 6/Figure 6f-uncropped scans of blots.jpg]
